# Use of the Chemcatcher® passive sampler and time-of-flight mass spectrometry to screen for emerging pollutants in rivers in Gauteng Province of South Africa

**DOI:** 10.1007/s10661-019-7515-z

**Published:** 2019-05-21

**Authors:** Cornelius Rimayi, Luke Chimuka, Anthony Gravell, Gary R. Fones, Graham A. Mills

**Affiliations:** 1 Department of Water and Sanitation, Resource Quality Information Services (RQIS), Roodeplaat, P. Bag X313, Pretoria, 0001 South Africa; 2 School of Chemistry, University of the Witwatersrand, P. Bag 3, Wits, Johannesburg, 2050 South Africa; 3 Natural Resources Wale, NRW Analytical Services, Swansea University, Faraday Building, Singleton Campus, Swansea, SA2 8PP UK; 4 School of Earth and Environmental Sciences, University of Portsmouth, Burnaby Road, Portsmouth, PO1 3QL UK; 5 School of Pharmacy and Biomedical Sciences, University of Portsmouth, White Swan Road, Portsmouth, PO1 2DT UK

**Keywords:** Chemcatcher®, Emerging pollutants, Pharmaceuticals and personal care products, Time-of-flight mass spectrometry, Screening, Surface water

## Abstract

Many rivers in urbanised catchments in South Africa are polluted by raw sewage and effluent to an extent that their ecological function has been severely impaired. The Hennops and Jukskei Rivers lying in the Hartbeespoort Dam catchment are two of the worst impacted rivers in South Africa and are in need of rehabilitation. Passive sampling (Chemcatcher® with a HLB receiving phase) together with high-resolution tandem mass spectrometry–targeted screening was used to provide high sensitivity and selectivity for the identification of a wide range of emerging pollutants in these urban waters. Over 200 compounds, including pesticides, pharmaceuticals and personal care products, drugs of abuse and their metabolites were identified. Many substances (~ 180) being detected for the first time in surface water in South Africa. General medicines and psychotropic drugs were the two most frequently detected groups in the catchment. These accounted for 49% of the emerging pollutants found. Of the general medicines, antihypertensive agents, beta-blocking and cardiac drugs were the most abundant (28%) classes detected. The Hennops site, downstream of a dysfunctional wastewater treatment plant, was the most polluted with 123 substances detected. From the compounds detected, peak intensity–based prioritisation was used to identify the five most abundant pollutants, being in the order caffeine > lopinavir > sulfamethoxazole > cotinine > trimethoprim. This work provides the largest available high-quality dataset of emerging pollutants detected in South African urban waters. The data generated in this study provides a solid foundation for subsequent work to further characterise (suspect screening) and quantify (target analysis) these substances.

**Electronic supplementary material:**

The online version of this article (10.1007/s10661-019-7515-z) contains supplementary material, which is available to authorized users.

## Introduction

Urban waters are under increasing environmental threat from emerging pollutants (EPs) originating from inputs of pharmaceuticals and personal care products (PPCPs) as well as household, agricultural and industrial chemicals (Archer et al. 2017a; Mueller et al. 2011). PPCPs are a large group of compounds that include over-the-counter medicines, prescription drugs as well as cleaning and personal hygiene products. Many PPCPs and other EPs are introduced into waterways through wastewater treatment plants with poor removal efficiencies, accidental spills and improper disposal of waste (Hernandez et al. 2015; Kaserzon et al. 2014; Madikizela et al. 2017a). The effect of EPs on aquatic organisms, humans and wildlife has not been fully elucidated, as the majority of PPCPs detected in surface water are usually below the concentrations that cause acute effects (Wong and MacLeod 2009). However, the presence of a plethora of EPs at low concentrations in heavily contaminated aquatic systems can have a synergistic effect. This can exert pharmacological and metabolic effects capable of altering homeostasis, physiological function and behaviour as well as phenotypic plasticity in aquatic animals (Reis-Santos et al. 2018; Saaristo et al. 2018). The impacts of new chemicals, which are continuously being developed and introduced to consumers worldwide, are a growing issue generating considerable interest amid environmental safety concerns (Ebele et al. 2017). Direct effects of different PPCPs on exposed fauna have been highlighted by Archer et al. (2017a).

A review of pharmaceuticals occurring in water bodies worldwide indicated that non-steroidal anti-inflammatory drugs (NSAIDs), antibiotics and carbamazepine occur widely in Europe, Hong Kong and the USA (Fekadu et al. 2019; Yang et al. 2017). Data for Africa shows antibiotics, antiepileptics, anti-malaria drugs, NSAIDs and steroid hormones are detected frequently in surface water bodies (Fekadu et al. 2019; Madikizela et al. 2017a). The antiretroviral drugs (lamivudine, nevirapine and zidovudine) and the antibiotics (ciprofloxacin, metronidazole, sulfamethoxazole and trimethoprim) have been detected frequently in Kenya (K'oreje et al. 2016; Ngumba et al. 2016). Analgesics and NSAIDs (acetaminophen, codeine, diclofenac and ibuprofen) and the anticonvulsant, carbamazepine, were often found in water in Cameroon (Branchet et al. 2019). However, numerous authors have stated that there is limited data available on the overall occurrence of EPs in Africa (Faleye et al. 2017; K'oreje et al. 2016; Madikizela et al. 2017a). Most studies to date characterised a few classes of compounds in surface or wastewater (Archer et al. 2017a; Agunbiade and Moodley 2016; Matongo et al. 2015a, b; Sorensen et al. 2015; Wood et al. 2015). The majority of studies on the occurrence of PPCPs in surface waters in South Africa were conducted in Gauteng and KwaZulu-Natal Provinces with fewer studies having been undertaken in Western Cape, Free State and Mpumalanga Provinces (Archer et al. 2017a). Antibiotics, antiepileptics, antiretrovirals, beta-blockers, NSAIDs, steroid hormones and caffeine were the most frequently detected PPCPs in South African waters (Archer et al. 2017a). Caffeine and the pharmaceuticals, acetaminophen, carbamazepine, diclofenac, efavirenz, ibuprofen, ketoprofen, naproxen, sulfamethoxazole and triclosan, were detected at high concentrations (< 0.2 to 19 μg L^−1^) in various rivers in KwaZulu-Natal Province (Agunbiade and Moodley 2014; Agunbiade and Moodley 2016; Matongo et al. 2015a, b; Madikizela and Chimuka 2016; Madikizela and Chimuka 2017a, b; Madikizela et al. 2014; Madikizela et al. 2017b; Mtolo et al. 2019; Sibeko et al. 2019). Concentrations of carbamazepine, methocarbamol and venlafaxine found in Gauteng and North West Provinces ranged between 0.001 and 0.094 μg L^−1^ (Rimayi et al. 2018a).

A reason that the range of contaminants present in environmental waters in South Africa has not been investigated thoroughly is the lack of high-resolution instrumentation and associated EP databases. The use of high-resolution mass spectrometry provides accurate mass spectra, enabling confident identification of pollutants (Alyzakis et al. 2018). Previously, this approach was used by K'oreje et al. (2012), to screen for the presence of 43 ‘priority’ pharmaceuticals in the Nairobi River basin in Kenya. Furthermore, workers to date have used low-frequency, low-volume and spot (bottle or grab) sampling regimes that make it difficult to detect episodic contamination incidents (Vrana et al. 2005; Lissalde et al. 2016; Rimayi et al. 2018a). The use of passive sampling devices (e.g. polar version of the Chemcatcher® or polar organic chemical integrative sampler (POCIS) alongside high-resolution mass spectrometry/mass spectrometry (HR-MS/MS) can overcome some of these difficulties. Guibal et al. (2015) and Soulier et al. (2016) used this approach by combining POCIS with HR-MS/MS for screening pollutants; the procedure was shown to have a better overall detection efficiency than the use of spot sampling.

Our study undertook a similar approach to qualitatively survey the range and frequency of occurrence of EPs in two of the most impacted rivers in Gauteng Province of South Africa. We used the Chemcatcher® (Mills et al. 2007, 2014) fitted with an Oasis® HLB-L (a copolymer of divinylbenzene and vinyl pyrrolidinone) solid-phase extraction disk as the receiving phase. This design of device is effective at sequestering a wide range of PPPCs in wastewater effluent (Petrie et al. 2016).

## Materials and methods

### Field sampling sites

**Fig. 1:**
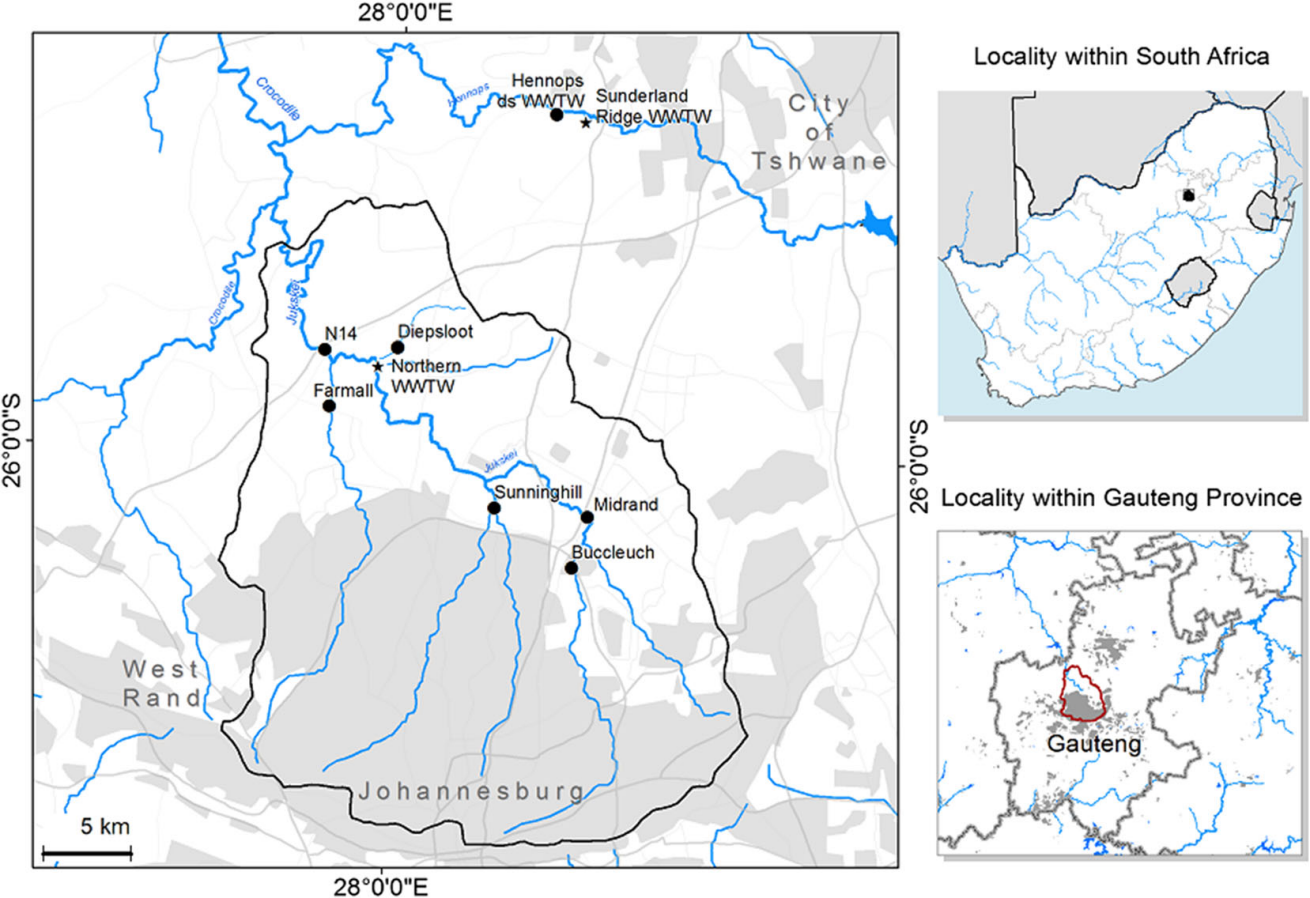
Sampling sites along (downstream) the Jukskei River (Buccleuch, Midrand, Sunninghill, Farmall, Diepsloot and N14 sites) and the Hennops River (Hennops ds). Further details are provided in Table S1

The Hennops and Jukskei Rivers form the major Hartbeespoort Dam catchment, supplying > 90% of the water into the Dam (Amdany et al. 2014a) (Fig. 1). Due to the impact of pollution in these two rivers, the Hartbeespoort Dam is in a chronic hypertrophic state (Hart and Matthews 2018). The Jukskei River has its source in the central industrialised Gauteng Province; parts of the river have pollutant concentrations approaching that of raw sewage, particularly in the dry season (Wimberley and Coleman 1993). Pollution in the Jukskei River is attributed to rapid urban population growth in informal settlements and to the Northern Wastewater Treatment Works (WWTW) which is located upstream of the N14 site (Rimayi et al. 2018a). The Jukskei River comprises four major tributaries each influencing water quality within the catchment. Five sampling sites (Fig. 1) were selected along the main tributaries of the river to assess the impacts of two different communities having vastly different lifestyles. Farmall and Sunninghill are sites that are least affected by domestic and industrial pollutants. These sites drain water from affluent suburban areas, with exceptional infrastructure, high-quality urban town planning and wastewater sewer systems on a par with the developed world. Buccleuch, Diepsloot and Midrand sites drain water from areas with poor populations, the majority who live in informal settlements with limited or no access to toilets and modern ablution facilities. Site N14 was selected as it lies 4 km downstream of the Northern WWTW discharge point and is also impacted by inputs of EPs further up the catchment. Further details of the six Jukskei field sites are provided in Table S1.

The Hennops River has its source on the eastern parts of Gauteng Province (Fig. 1) and has one major point source of contamination, the Sunderland Ridge WWTW. The Hennops downstream (ds) site was located 200 m downstream of the Sunderland Ridge WWTW. This work frequently spews large quantities of untreated wastewater into the river. As the Hennops River passes from the suburbs of Kempton Park and into the townships of Tembisa and Ivory Park, the surface water resembles sewage in central parts of the Centurion town as it meets the effluent from the Sunderland Ridge WWTW (Fig. 1).

### Total dissolved solids and dissolved oxygen

Total dissolved solids (TDS) and dissolved oxygen (DO) were measured using a calibrated YSI multi-parameter meter (Model 556, YSI Inc., Yellow Springs, OH, USA). After in-field verification of the dissolved oxygen calibration, the probe was placed in the water at the site where the Chemcatcher® was deployed. Measurements were taken during deployment and retrieval of the sampler.

### Preparation and deployment of Chemcatcher®

Three components, PTFE Atlantic Chemcatcher® bodies were manufactured by AT Engineering Ltd. (Tadley, Hampshire, UK) (Fig. S1). Prior to use, components were washed by soaking overnight in a 5% Decon 90 (Decon Laboratories Ltd., Hove, UK) detergent solution and rinsed with ultrapure water (ELGA Purelab Ultra, Marlow, UK). This was followed by washing in acetone (ultrasonic bath for 10 min), removal, rinsed with water and dried. A Horizon Atlantic™ (Oasis® hydrophilic-lipophilic balanced (HLB-L)) disk (47 mm diameter) (Labmedics Ltd., Abingdon, UK) was used as the sampler receiving phase. This was soaked in methanol overnight and then activated by drawing 50 mL methanol (HPLC grade, Fisher Scientific, UK) followed by 100 mL water (HPLC grade, Fisher Scientific, UK) through the disk under gentle vacuum. In order to prevent the disks from drying out, the HLB-L disks were left submerged in ultrapure water until assembly. Polyethersulfone (PES) (Supor® 200, 0.2 μm pore diameter; punched out to 52-mm diameter disks) (Pall Europe Ltd., Portsmouth, UK) was used as the diffusion-limiting membrane. PES membranes were washed to remove any excess oligomers from the manufacturing process by soaking overnight in methanol, rinsing with water and keeping wet until use. The sampler was made by placing a HLB-L receiving phase disk rough side down onto the Chemcatcher® supporting plate followed by a preconditioned PES membrane. It was important that no air bubbles remained between the two surfaces. The Chemcatcher® retaining ring was used to secure the disk and membrane in place. Prior to deployment, the assembled devices were kept submerged in ultrapure water. Before taking any devices to the field, the PTFE lid was fitted ensuring that there was a small quantity of water remaining in the top well and then secured.

A bespoke rig was used to deploy the samplers at the field sites to ensure that they remained submerged throughout the trial (Fig. S2). This comprised a Perspex® sheet (250 mm × 250 mm × 3 mm thick) from which two Chemcatcher® samplers were fixed with the PES membrane facing downwards in the water column. Devices were deployed at all sites for 14 days, between 17 September and 1 October, 2017 at a depth of 30–40 cm below the surface of the water. With the exception of the Hennops ds site, which recorded a negligible flow, all other sites recorded a water flow rate of at least 0.45 m^3^ s^−1^. The deployment rigs were stable under these flow conditions. After retrieval, the Chemcatcher® assemblies were resealed with the PTFE transport lid, ensuring that a small quantity of water remained in the top well of the device. During each deployment and retrieval operation, a field blank sampler was exposed and then resealed and handled subsequently as for the field-exposed devices. Samplers were immediately transported (cool boxes at 4 °C) to the Department of Water and Sanitation Laboratory (Pretoria, South Africa) for further processing. Any surface biofouling of the sampler body was removed gently using a soft brush. Chemcatcher® devices were then placed into individually labelled zip-lock polyethylene bags, packed into a cool box (< 10 °C) and couriered overnight to the Natural Resources Wales (Swansea, UK) laboratory for instrumental analysis.

### Extraction of HLB-L disks

In the laboratory, the Chemcatcher® samplers were dissembled, the HLB-L receiving phase disk removed and the PES membrane discarded. Prior to extraction, the HLB-L disks were allowed to dry on solvent-rinsed aluminium foil (24 h at room temperature). EPs were then eluted from the disks (methanol, 40 mL) under gravity using a glass extraction funnel manifold. Methanol was collected into pre-cleaned glass vials (60 mL). The extracts were evaporated (Genevac EZ-2 centrifugal rotary evaporator, Genevac Ltd., Ipswich, UK) to near dryness with the evaporator set at a low boiling point mode. Each extract was reconstituted with methanol (0.5 mL) before transferring to a vial (2 mL) and adding a further aliquot of methanol (0.5 mL). The extracts were further diluted (10×) using mobile phase B (see below) before instrumental analysis.

### High-resolution mass spectrometry

A Dionex Ultimate 3000 UHPLC (Thermo Fisher Scientific, Bremen, Germany) instrument interfaced to a Bruker Maxis Impact II electrospray high-resolution time-of-flight tandem mass spectrometer (Q-ToF-MS) (Bruker Daltonics, Bremen, Germany) with Bruker HyStar acquisition software (rev. 3.2) was used for instrumental analysis. A Dionex Acclaim RSLC 120 C_18_ analytical column (2.1 i.d. × 100 mm length, 2.2 μm particle size, Thermo Fisher Scientific, Dreieich, Germany) and a Waters VanGuard, Acquity UPLC BEH C_18_ 1.7 μm particle size, (Dublin, Ireland), guard column was used to separate the compounds. A 20-μL injection volume of extract was used.

The Q-ToF-MS was equipped with an electrospray ionisation source, operating in positive ionisation mode. Mobile phase was (A) methanol with 5 mM ammonium formate and 0.01% *v/v* formic acid and (B) an aqueous solution comprised of 10% of methanol, 5 mM ammonium formate and 0.01% formic acid. The gradient and flow elution programme was 0 min, 1% B, 0.2 mL min^-1^; 3 min, 39% B, 0.2 mL min^-1^; 14 min, 99.9% B, 0.4 mL min^-1^; 16 min, 99.9% B, 0.48 mL min^-1^; 16.1 min, 1% B, 0.48 mL min^-1^; 19.1 min, 1% B, 0.2 mL min^-1^; and 20 min, 1% B, 0.2 mL min^-1^.

The Q-ToF-MS–operating parameters were capillary voltage, 2500 V; end plate offset, 500 V; nebulizer pressure, 2 bar (N_2_); drying gas, 8 L min^-1^ (N_2_); and drying temperature, 200 °C. The Q-ToF-MS system was used in broadband collision-induced dissociation (bbCID) acquisition mode and recorded spectra over the range 30−1000 Da at a scan rate of 2 Hz. The Bruker ‘bbCID’ mode provided MS and MS/MS spectra at the same time, whilst working at two different collision energies. Low collision energy of 6 eV was used to acquire MS spectra, and a higher energy setting of 30 eV was used to obtain MS/MS spectra. The higher energy setting was ramped from 80 to 120% of its value (i.e. from 24 to 36 eV). Data were collected by the mass spectrometer between 0.1 and 15.0 min.

A mass axis calibration was undertaken at the beginning of every chromatographic run by infusing a mixture of 1 mM sodium formate in water/isopropanol/formic acid (1:1:0.01 *v/v/v*) with a syringe pump into the mass spectrometer ahead of the elution of the first target compound from the analytical column.

Calibration of the acquired sample data files was performed using the high-precision algorithm of the instrument. Target compounds (~ 2,500 substances included in the PesticideScreener™ 2.1 and ToxScreener™ 2.1 libraries) were identified in the solvent extracts obtained from the Chemcatcher® based on the retention time, mass accuracy, isotopic pattern and diagnostic MS/MS fragments. The extracted ion chromatograms of all the compounds (including protonated and sodiated molecular ions together with their associated fragment ions) were produced automatically using Bruker Target Analysis for Screening and Quantitation (TASQ)® 1.4 software. These were assessed against the following limits for all compounds in the two Bruker databases: ± 5 ppm for mass accuracy, isotopic fit < 250 (expressed as mSigma) and ± 0.5 min for the retention time tolerance. An example of the analytical workflow is given in Figs. S3 and S4. The detection of at least one product ion for each precursor ion was mandatory. Manual evaluation of the data was undertaken where necessary. The libraries were, however, not exhaustive of all the compounds that could be found in the sample extracts. No attempt was made to further identify such compounds manually using untargeted screening approaches.

## Results and discussion

### Total dissolved solids and dissolved oxygen

TDS (a measure of the inorganic salts or electrically charged dissolved cations and anions in water) and DO were used as an index of the extent of water pollution at the sampled field sites. According to the World Health Organization, water with a TDS < 0.30 g L^−1^ is considered good (WHO 2003). The highest TDS were at the Hennops ds site at deployment (1.02 g L^−1^) and retrieval (0.67 g L^−1^) of the Chemcatcher® devices. The lowest TDS were at the Sunninghill site, at deployment (0.26 g L^−1^) and at retrieval (0.27 g L^−1^) of the samplers (Table S2). A DO content < 5.00 mg L^−1^ does not adequately support aquatic life and causes stress in aquatic fauna (WHO 2003). The lowest DO measurement was at the Hennops ds site with 0.57 mg L^−1^ on retrieval (with a higher DO of 5.18 mg L^−1^ on deployment). This difference in the DO values can be explained by the frequent release of untreated sewage from the Sunderland Ridge WWTW. The Diepsloot site recorded a low DO on deployment (3.09 mg L^−1^) and on retrieval (2.00 mg L^−1^). Likewise, the Buccleuch site also had low DO values (Table S2). The extent of pollution at these three sites is that the water quality appears similar to untreated sewage. As expected, the Sunninghill site recorded the highest DO values (~ 9 mg L^−1^), indicating a better water quality and was the least-contaminated site. The pH for all the sites was ~ neutral, ranging between 6.78 and 7.26 and the temperature of the water at the sites ranged from 14.2 to 14.6 °C.

### Chemcatcher® passive samplers

The Chemcatcher® samplers withstood the harsh environmental conditions during the 2-week field deployments. Those devices that were exposed at the sites where there was untreated sewage present had some degree of surface biofouling present on the PES membrane. The Oasis® HLB-L sorbent used as the receiving phase can sequester a wide range of semi-polar and polar compounds (Petrie et al. 2016). This sorbent has been extensively used as receiving phase for both the polar Chemcatcher® and POCIS passive samplers (Petrie et al. 2016; Castle et al. 2018a, b; Iparraguirre et al. 2017; Magi et al. 2018). It should be noted, however, that the HLB-L sorbent only has a limited capacity to efficiently extract highly ionic substances such as anionic (e.g. diclofenac, ibuprofen and naproxen, that are widely used as NSAIDs) (Lindqvist et al. 2005) and cationic drugs (e.g. various cathinones) (Bade et al. 2017; Gonzalez-Marino et al. 2016) that have previously been reported to be present in wastewaters, including those in South Africa (Agunbiade and Moodley 2016; Gumbi et al. 2017). The use of another type of receiving phase in the Chemcatcher® (e.g. ion-exchange sorbents) could be used to overcome this issue (Townsend et al. 2018). Methanol is a suitable extraction solvent and can recover ~ 100% of the sorbed analytes (Petrie et al. 2016; Castle et al. 2018a, b).

Furthermore, some of the more non-polar PPCP type pollutants, e.g. the antibacterial and antifungal agent triclosan (predicted log *K*_ow_ ~ 5) can have a high affinity to bind to the PES membrane and, therefore, may not be found to be present in the receiving phase of the sampler (Kaserzon et al. 2014). The PES membrane was not extracted and analysed in our study. The use of a bound sorbent in the form of a 47-mm disk is also advantageous, as the material cannot move during field deployment. Hence, the active sampling area of the device remains constant and yields more reproducible data. This has been reported to be an issue with the use of the loose powder in the POCIS (Mills et al. 2014). For this initial investigation, we only screened for the presence of polar pollutants to provide baseline data for future studies. We did not use estimated Chemcatcher® sampler uptake rates (i.e. *R*_*s*_) values (Petrie et al. 2016) for PPCPs and metabolites to attempt semi-quantify the time-weighted average concentrations the compounds detected. This will be a subject of follow-up work.

Using the Chemcatcher®, a large number (219 compounds) and range of EPs were identified from the seven sites. The exposed field blanks only contained trace amounts of the commonly used insect repellent DEET. There were several other extracted ion chromatographic peaks; however, the identity of these compounds could not be assigned with certainty using the prescribed analytical workflow. There was good reproducibility in the data obtained between the duplicate samplers at the same sites. The identified compounds included pharmaceuticals, drugs of abuse and their metabolites, pesticides and food additives. Several of these substances (i.e. benzododecinium, fluconazole, ephedrine, griseofulvin, guaifenesin, metformin, pseudoephedrine, practolol, tramadol and trimethoprim) were not previously considered widespread in surface waters in South Africa.

### Classification of emerging pollutants

Most previous studies, to measure EPs, particularly pharmaceuticals, in inland and coastal waters in South Africa have used a quantitative and targeted approach (Madikizela et al. 2017a, Petrik et al. 2017). In order to simplify the data generated, several workers have proposed different ways to classify the various classes of EPs detected in surface water (Reis-Santos et al. 2018; Archer et al. 2017b; Naude et al. 2015). We classified the compounds into six main groups (medicines, psychotropic drugs, metabolites, central nervous system (CNS) stimulants, poisons and food components/additives) and 37 subgroups that describe their major uses (Tables 1, 2, 3, 4, 5 and 6). For this study, a distinction was drawn between medicines, psychotropic drugs and CNS stimulants, based on their major form of use in South Africa.

**Table 1:** Medicines detected in the Hennops and Jukskei Rivers, together with their registration status with the South African Health Products Regulatory Authority (SAHPRA, as of 30 October 2018) and scheduling

Type	Emerging pollutant	*Reg	**Scheduling	Hennops ds	N14	Diepsloot	Farmall	Sunninghill	Midrand	Buccleuch
AL and PK	Galantamine	Y	S4	X	X	X	X	√	X	X
	Memantine	Y	S4	√	√	X	X	X	X	X
	Rivastigmine	Y	S5	X	X	X	√	X	X	X
	Ropinirole	Y	S4	X	X	X	X	√	X	X
	Sulfasalazine	N	N/A	X	X	X	X	X	√	√
ANHIS	Cetirizine	Y	S2	√	√	√	√	√	X	√
	Chlorpheniramine	Y	S2	√	√	√	X	X	√	X
	Diphenhydramine	Y	S2	√	√	√	√	X	√	X
	Fexofenadine	Y	S2	√	√	X	√	√	√	√
	Loratadine	Y	S2	X	√	X	X	X	X	X
	Orphenadrine	Y	S2	√	X	X	X	X	X	X
	Promethazine	Y	S2	√	X	X	X	X	X	X
ANSTH	Bupivacaine	Y	S4	√	X	X	X	√	X	X
	Caproylresorcinol	N	N/A	X	X	X	√	X	X	X
	Ketamine	Y	S5	X	√	X	X	√	X	X
	Lidocaine	Y	S4	√	√	X	√	√	√	√
	Prilocaine	Y	S1	X	X	X	√	X	X	X
	Procaine	Y	S1	X	X	√	X	X	X	X
ANTB	Azithromycin	Y	S4	√	√	√	X	√	√	√
	Erythromycin	Y	S4	√	X	X	X	X	X	X
	Nalidixic acid	Y	S4	√	√	X	X	X	X	X
	Sulfabenzamide	N	N/A	X	√	X	√	X	√	X
	Sulfamethoxazole	Y	S4	√	√	√	√	√	√	√
	Sulfapyridine	N	N/A	√	√	X	√	√	√	√
	Trimethoprim	Y	S4	√	√	√	√	√	√	√
ARV	Lopinavir	Y	S4	√	√	X	√	√	√	√
	Ritonavir	Y	S4	√	√	X	√	√	√	√
ASTHM	Carbuterol	N	N/A	√	X	√	√	X	X	X
	Hydrocortisone	Y	S4	X	X	X	X	X	X	√
	Salbutamol	Y	S2,S3 and S4	√	X	X	X	X	X	X
	Theophylline	Y	S2	X	X	X	X	X	√	√
CAN	Aminoglutethimide	N	N/A	√	X	X	X	X	√	√
	Bicalutamide	Y	S4	X	√	X	X	X	X	X
CHOL	Bezafibrate	Y	S3	√	X	√	X	X	√	√
	Lovastatin	Y	S4	X	X	√	X	X	X	X
	Rosuvastatin	Y	S4	√	X	X	X	X	X	X
CMED	Dextromethorphan	Y	S2	√	√	X	X	X	X	X
	Guaifenesin	Y	S2	√	√	√	√	√	√	√
	Theophylline	Y	S2	X	√	X	X	X	X	X
DIAB	Metformin	Y	S3	√	√	√	√	√	√	√
	Sitagliptin	Y	S3	X	√	X	X	X	X	X
	Vildagliptin	Y	S3	X	X	X	√	√	√	√
FLU	Amantadine	Y	S4	√	√	X	√	√	√	X
	Norephedrine	Y	S2	√	X	X	√	√	√	√
	Pseudoephedrine	Y	S2	√	√	√	√	√	√	√
	Salbutamol	Y	S2 and S3	X	√	X	X	X	X	X
	Theophylline	Y	S2	√	X	X	X	X	X	X
HHBB	Adenosine	Y	S4	√	√	√	√	√	√	√
	Ajmaline	N	N/A	X	X	X	X	X	X	√
	Amiloride	Y	S3	√	√	√	X	X	X	√
	Amrinone	N	N/A	√	X	X	X	X	X	X
	Apophedrin	N	N/A	√	√	X	X	X	X	X
	Atenolol	Y	S3	√	√	√	√	√	√	√
	Atropine	Y	S2, S3, S4 and S5	X	X	X	X	√	√	X
	Bisoprolol	Y	S3 and S4	√	√	X	√	√	X	√
	Celiprolol	N	N/A	X	X	X	X	X	√	X
	Clopidogrel	Y	S3	√	√	X	X	√	X	X
	Diltiazem	Y	S3	√	√	X	X	X	X	X
	Disopyramide	Y	S4	X	X	X	X	√	X	X
	Ephedrine	Y	S2 and S3	√	√	√	√	√	√	√
	Esmolol	N	N/A	X	X	X	X	√	√	√
	Etilefrine	Y	S2	X	X	X	X	√	√	√
	Flecainide	Y	S4	√	√	√	√	√	X	X
	Hydrochlorothiazide	Y	S3	√	√	X	X	√	X	X
	Indapamide	Y	S3	√	X	X	X	X	X	X
	Irbesartan	Y	S3	√	√	X	√	X	√	√
	Isoxsuprine	N	N/A	X	X	√	X	X	X	X
	Losartan	Y	S3	√	√	X	√	√	√	√
	Metipranolol	Y	S3	X	X	X	X	X	√	X
	Practolol	N	N/A	√	√	√	√	√	√	√
	Propranolol	Y	S3	√	√	X	X	√	X	X
	Rivaroxaban	Y	S4	√	√	X	X	X	X	X
	Sotalol	Y	S3	√	√	X	√	√	√	√
	Telmisartan	Y	S3	√	√	X	√	√	√	√
	Temazepam	Y	S5	X	X	X	X	√	X	X
	Valsartan	Y	S3	√	√	X	√	√	√	√
	Verapamil	Y	S3	X	√	X	X	X	X	X
HOR	Corticosterone	N	N/A	X	X	√	X	X	X	X
	Cortisone	N	N/A	X	X	√	X	X	X	X
	Melatonin	Y	S4	√	X	X	X	√	X	√
	Progesterone	Y	S4	X	X	X	√	X	X	√
LAX	Bisacodyl	N	N/A	X	X	X	X	√	X	√
MALD	Mefloquine	Y	S4	√	X	X	X	X	X	X
	Proguanil	Y	S2, S3 and s4	√	√	X	√	√	√	√
	Quinine	Y	S2 and S4	X	X	√	X	X	X	X
	Sulfadoxine	Y	S4	X	√	X	X	X	X	X
MRELX	Aceclidine	Y	S4	X	X	X	X	X	√	X
	Methocarbamol	Y	S2	√	√	√	√	√	√	√
	Orphenadrine	Y	S2	X	√	√	X	X	X	X
	Pholedrine	N	N/A	X	√	√	X	X	X	X
NAU	Codeine	Y	S2, S3 and S5	X	X	X	X	√	√	√
	Cyclizine	Y	S2	√	√	X	√	X	X	X
NSAID	Diclofenac	Y	S1–S4	√	√	√	√	√	√	√
	Ibuprofen	Y	S1, S2 and S3	√	X	√	√	X	√	X
	Ketoprofen	Y	S1 and S3	√	√	X	X	X	X	X
	Mefenamic acid	Y	S2 and S3	√	√	X	√	X	√	X
	Mefexamide	N	N/A	X	X	X	X	X	√	X
	Naproxen	Y	S1–S4	√	√	√	√	√	√	√
	Phenazone	Y	S1 and S2	√	X	√	X	X	√	X
PK	Acetaminophen	Y	S1–S5	X	√	√	√	√	√	√
	Phenacetin	N	N/A	X	√	√	√	X	√	√
	Salicylamide	Y	S1 and S2	√	√	√	√	√	√	√
PSYM	2 C-D	N	N/A	√	X	X	X	X	X	X
	Amisulpiride	N	N/A	X	√	X	X	X	√	√
	Amitriptyline	Y	S5	√	√	X	X	X	X	X
ULC	Cimetidine	Y	S2 and S3	√	X	√	X	X	X	√
	Omeprazole	Y	S2 and S4	X	√	X	X	X	X	X
SEXD	Flibanserin	N	N/A	X	√	X	X	X	X	X
*Total number detected*		*86*		*64*	*61*	*34*	*42*	*45*	*48*	*46*

*Registration with the South African Health Products Regulatory Authority (SAHPRA, as of 30 October 2018)

**See Table S3 for schedule description

*AL&PK* Alzheimer’s disease, arthritis and Parkinson’s disease drug, *ANHIS* antihistamine medication, *ANSTH* anaesthetic drug, *ANTB* antibiotic drug, *ARV* antiretroviral drug, *ASTHM* asthma drug, *CAN* cancer drug, *CHOL* hypercholesterolemia (cholesterol) drug, *CMED* cough medicine, *DIAB* diabetes drug, *FLU* influenza medication, *2 C-D* 2,5-dimethoxy-4-methylphenethylamine, *CHBB* cardiac and antihypertensive agent/beta-blocking drug, *HOR* hormone, *LAX* laxative, *MALD* malaria drug, *MRELX* muscle relaxant, *NAU* nausea drug, *NSAID* nonsteroidal anti-inflammatory drug, *PK* pain killer, *PSYM* antipsychotic medication, *SEXD* hypoactive sexual desire disorder (HSDD) drug, *ULC* peptic ulcer medication

**Table 2:** Psychotropic drugs detected in the Hennops and Jukskei Rivers, together with their registration status with the South African Health Products Regulatory Authority (SAHPRA, as of 30 October 2018) and scheduling

Type	Emerging pollutant	*Reg	**Scheduling	Hennops ds	N14	Diepsloot	Farmall	Sunninghill	Midrand	Buccleuch
ANTD	Bupropion	Y	S5	X	√	X	X	X	X	X
	Citalopram	Y	S5	√	√	X	√	√	√	√
	Fluoxetine	Y	S5	√	√	X	X	X	X	X
	Maprotiline	Y	S5	√	√	X	X	X	X	X
	Moclobemide	Y	S5	X	√	X	X	X	X	X
	Norcitalopram	N	N/A	X	X	X	√	X	X	X
	Sertraline	Y	S5	√	√	X	X	X	X	X
	Venlafaxine	Y	S5	√	√	X	√	√	√	√
CANNB	Tetrahydrocannabinol	N	N/A	X	X	X	X	X	X	√
OPAN	Dihydrocodeine	Y	S6	√	√	X	X	X	X	X
	Hydrocodone	N	N/A	√	√	√	√	√	√	√
	Hydromorphone	Y	S6	√	√	√	X	X	√	√
	Ketobemidone	N	N/A	X	X	√	X	√	X	X
	Meptazinol	N	N/A	X	X	X	X	√	X	X
	Morphine	Y	S6	√	√	√	X	X	√	√
	Oxycodone	Y	S6	√	√	X	√	√	X	X
	Oxymorphone	N	N/A	X	X	X	√	X	X	X
	Pethidine	Y	S6	X	X	X	√	X	X	X
	Tramadol	Y	S5	√	√	√	√	√	√	√
PSYSTIM	5-Methoxytryptamine	N	N/A	√	√	X	X	X	X	X
	5-MeOT	N	N/A	X	X	X	X	√	X	X
	6-APB	N	N/A	X	√	X	X	X	X	X
	Amphetamine	N	N/A	X	√	X	√	X	X	X
	AMT	N	N/A	X	√	X	X	X	X	X
	bk-MDDMA	N	N/A	X	√	X	X	√	X	√
	Cathinone	N	N/A	X	X	X	X	X	X	√
	Ethylone	N	N/A	X	√	X	X	X	X	X
	MDA	N	N/A	X	X	√	X	X	X	X
	MDAI	N	N/A	X	X	X	X	X	X	√
	MDEA	N	N/A	X	X	X	X	√	√	√
	MDMA	N	N/A	X	X	X	X	√	X	X
	Methamphetamine	N	N/A	X	√	√	X	√	√	√
	Methcathinone	N	N/A	√	X	X	X	X	√	√
	Phentermine	Y	S5	√	√	X	X	√	√	√
	Pyrovalerone	N	N/A	X	X	X	X	√	X	X
	Sulpiride	Y	S5	X	X	X	X	√	√	√
EPD	Carbamazepine	Y	S3 and S5	√	√	√	√	√	√	√
	Gabapentin	Y	S3	√	X	X	√	X	X	X
	Lamotrigine	Y	S3	√	√	√	√	√	√	√
	Levetiracetam	Y	S3	√	√	X	√	X	√	√
	Nordiazepam	N	N/A	√	√	X	X	X	X	X
	Oxcarbazepine	Y	S3	√	√	√	√	√	X	√
	Phenytoin	Y	S3	X	X	√	√	X	X	X
	Pregabalin	Y	S5	√	X	X	X	X	X	X
	Primidone	Y	S3	X	√	√	√	X	X	X
SED/T	Clobazam	Y	S5	√	√	X	X	X	X	X
	Meprobamate	Y	S5	√	√	√	√	√	√	√
	Methaqualone	N	N/A	√	√	√	√	√	√	√
	Oxazepam	Y	S5	√	√	X	√	√	√	√
	Promazine	N	N/A	√	X	X	X	X	X	X
	Sulpiride	Y	S5	√	√	X	X	X	X	X
	Temazepam	Y	S5	√	√	X	X	X	√	X
*Total number detected*				*28*	*33*	*14*	*19*	*21*	*18*	*22*

*Registration with the South African Health Products Regulatory Authority (SAHPRA, as of 30 October 2018)

**See Table S3 for schedule description

*5-MeOT* 6-(2-aminopropyl)benzofuran), *6-APB* alpha-pyrrolidinopentiophenone, *AMT* alpha-methyltryptamine, *MDA* methylenedioxyamphetamine, *bk-MDDMA* dimethylone, *MDAI* 5,6-methylenedioxy-2-aminoindane, *MDEA* 3,4-methylenedioxy-N-ethylamphetamine, *MDMA* 3,4-methylenedioxy-methamphetamine, *ANTD* antidepressant, *CANNB* cannabis, *OPAN* opioid analgesic, *PSYSTIM* psychoactive stimulant, *EPD* epilepsy and anticonvulsant drug, *SED/T* sedative/tranquiliser drug

**Table 3:** Metabolites detected in the Hennops and Jukskei Rivers

Emerging pollutant	Hennops ds	N14	Diepsloot	Farmall	Sunninghill	Midrand	Buccleuch
10-Hydroxycarbamazepine	√	√	X	√	X	X	X
1-Hydroxymidazolam	√	√	X	√	√	√	√
3-Methylnorfentanyl	√	X	X	X	X	X	X
4-Acetamidoantipyrine (4-AAA)	√	√	X	√	√	√	X
4-Formylaminoantipyrine (4-FAA)	√	√	X	√	√	√	X
Acetaminodantrolene	X	X	X	√	X	X	X
Anabasine	X	X	√	X	X	√	X
Benzoylecgonine	√	√	√	√	√	√	√
Cotinine	X	X	√	√	√	√	√
Deacetyldiltiazem	X	√	X	X	X	X	X
Ecgonine methyl ester	√	X	X	√	X	√	X
HHMA	X	X	X	X	X	√	X
Hydroxycotinine	√	X	X	X	X	X	X
Norcitalopram	X	X	X	X	√	X	X
Norcocaine	√	√	√	√	√	√	√
Norcodeine	√	√	X	√	√	√	X
Nortramadol	X	X	X	X	X	X	√
Norvenlafaxine	X	√	X	X	√	X	X
O-Desmethylnortramadol	√	√	√	√	√	√	√
O-Desmethyltramadol	√	√	√	√	√	√	√
O-Desmethylvenlafaxine	√	√	√	√	√	√	√
Ritalinic acid	√	√	X	√	√	√	X
THC-OH. 11-OH-THC	X	X	X	X	√	X	X
*Total number detected*	*14*	*13*	*7*	*14*	*14*	*14*	*8*

**Table 4:** CNS stimulants detected in the Hennops and Jukskei Rivers

Type	Emerging pollutant	Hennops ds	N14	Diepsloot	Farmall	Sunninghill	Midrand	Buccleuch
AAS	Nandrolone	X	X	√	X	X	X	X
CNS STIM	2-Phenethylamine	√	√	√	√	√	√	√
	4-MePPP	√	√	X	X	X	X	X
	4-Methylbuphedrone	X	X	X	√	X	X	X
	Aceclidine	√	X	√	X	X	X	X
	Alpha-PVP	X	X	X	√	X	X	X
	Caffeine	√	√	√	√	√	√	√
	Caproylresorcinol	X	X	X	√	X	X	X
	Cathine	√	X	X	√	√	X	X
	Cocaine	X	√	X	√	√	√	√
	Ethylcathinone	√	X	X	X	X	X	X
	Hordenine	X	X	X	X	X	√	√
	Methedrone	X	X	√	X	X	X	X
	Methylphenidate	X	√	X	X	X	X	X
	Nicotine	√	√	√	X	X	√	√
	Pemoline	X	X	X	X	X	X	√
*Total number detected*		*7*	*6*	*6*	*7*	*4*	*5*	*6*

*AAS* androgen and anabolic steroid, *CNS STIM* central nervous system stimulant, *4-MePPP* 4′-methyl-α-pyrrolidinopropiophenone, *Alpha-PVP* alpha-pyrrolidinopentiophenone

**Table 5:** Pesticides and disinfectants detected in the Hennops and Jukskei Rivers

Type	Emerging pollutants	Hennops ds	N14	Diepsloot	Farmall	Sunninghill	Midrand	Buccleuch
ANT/DIS	Benzododecinium	√	√	√	√	√	√	√
Fungicide	Fluconazole	√	√	√	√	√	√	√
	Griseofulvin	√	√	√	√	√	√	√
	Propiconazole	√	√	√	X	X	√	√
	Tolnaftate	X	X	√	X	X	X	X
Herbicide	Prometryn	X	√	√	X	X	X	X
	Sebuthylazine	√	√	√	X	√	X	X
Insecticide	DEET	√	√	√	√	√	√	√
	Malathion	X	X	√	X	X	√	√
Rodenticide	Pyranocoumarin	X	X	X	X	√	X	X
Vermicide	Albendazole	X	X	√	X	X	X	X
*Total number detected*		*6*	*7*	*10*	*4*	*6*	*7*	*6*

*DEET N.N*-diethyl-m-toluamide, *ANT/DIS* antiseptic/disinfectant

**Table 6:** Food components and additives detected in the Hennops and Jukskei Rivers

Type	Emerging pollutants	Hennops ds	N14	Diepsloot	Farmall	Sunninghill	Midrand	Buccleuch
Amino acid	Tyramine	X	X	√	X	X	√	√
AS	Cyclamic acid	√	X	√	X	X	√	X
Chilli	Capsaicin	X	√	√	X	X	√	√
Choc	Theobromine	√	√	X	√	X	√	√
Spice	Harman	√	√	X	√	√	√	√
Vitamin	Biotin	X	X	√	X	X	X	X
	Nicotinamide	√	√	X	√	√	√	√
	Pyridoxine	X	X	√	X	X	X	X
WLPED	DMAA	X	√	X	X	X	X	X
*Total number detected*	*4*	*5*	*5*	*3*	*2*	*6*	*5*	

*AS* artificial sweetener, *Chilli* component of chillies, *Choc* chocolate component, *DMAA* dimethylamylamine, *WLPED* weight loss and performance-enhancing drug

It should be noted that some of the active pharmaceutical ingredients detected have multiple uses traversing more than one classification. For example, ephedrine is both a medicine and a CNS stimulant, but is also widely considered an illicit drug precursor. Many opioid analgesics (e.g. codeine and morphine) can be classified as both medicine and as psychotropic drugs of abuse (Archer et al. 2017b). Some CNS stimulants (e.g. ethylone) were originally for therapeutic uses but now can also be considered designer drugs of abuse (Zanda and Fattore 2017). The term medicine is broad and can be defined as any drug product that can be dispensed using a prescription or purchased over-the-counter (Morgan et al. 2017). Medicines are usually comprised of single or a combination of active pharmaceutical ingredients (Sanderson and Thomsen 2007). Psychotropic drugs have been defined as drugs that are capable of affecting the mind, emotions and behaviour (Matson and Neal 2009). Illicit drugs, antidepressants, anxiolytics, sedatives, antipsychotics, mood stabilisers and antiepileptic medications comprise the main categories of psychotropic medications (Stephenson et al. 2013).

### Trends of EPs detected in the Hartbeespoort Dam catchment

**Fig. 2:**
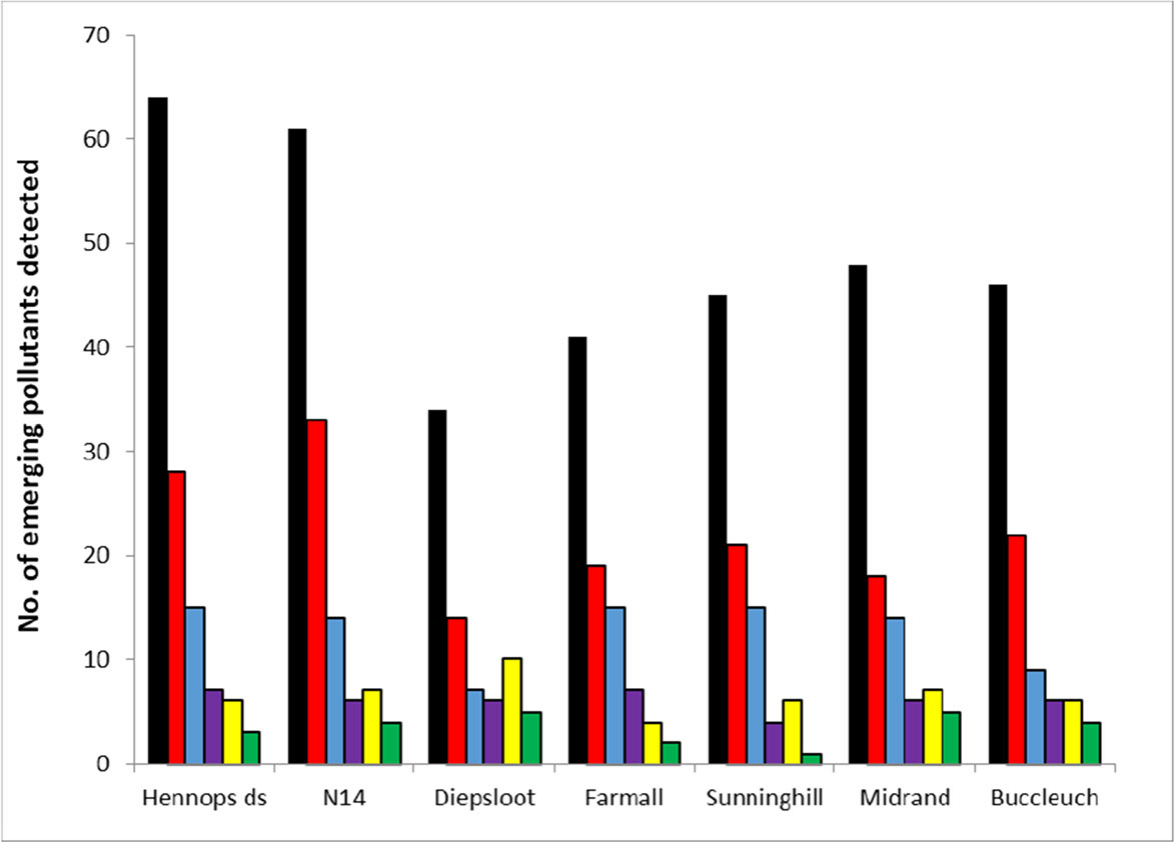
Classification and number of emerging pollutants detected in the Hennops and Jukskei catchment. Key: medicines (black box), psychotropic drugs (red box), metabolites (blue box), CNS stimulants (violet box), pesticides (yellow box) and components/additives (green box)

The overall abundance of EPs detected at the seven sampling sites at the Hartbeespoort Dam catchment was in the order of medicines (108 compounds) > psychotropic drugs (52) > metabolites (23) > CNS stimulants (16) > pesticides (11) > food components/additives (9). Medicines accounted for 49% of the compounds detected. The highest detections of 64 medicines were at the Hennops ds site, and the lowest detection of 34 medicines was at the Diepsloot site (Fig. 2). Medicines detected in the Jukskei river were in the order of N14 (61 compounds) > Midrand (48) > Buccleuch (46) > Sunninghill (45) > Farmall (42) > Diepsloot (34). The N14 site being located furthermost upstream was expected to be the most polluted.

Psychotropic drugs were the second most abundant (22%) class detected with many of them being drugs of abuse. The highest detection was recorded at the N14 site and the lowest detection at the Diepsloot site. Pesticides accounted for 7% of the EPs detected, with the highest detection rate at the Diepsloot site. CNS stimulants accounted for 6% of the EPs detected and were found at approximately the same rate at all sites (Fig. 2). Food components and additives were detected at the lowest rate in the catchment, accounting for only 3% of the EPs detected.

### Medicines detected in the catchment

Eighty percent of the medicines detected in the catchment was registered with the South African Health Products Regulatory Authority (SAHPRA) as of 30 October 2018 (Table 1), indicating that they can be dispensed legally by pharmacies and shops in South Africa. The most abundant medicines (28%) were cardiac, antihypertensive agents and beta-blocking (CHBB) drugs (Fig. S5). Beta-blockers are widely used to treat ischemic heart disease (Andreasen and Andersson 2018), hence their widespread use. The next most commonly detected classes included antihistamines, antibiotics and NSAIDs. All other medication classes accounted for < 5% of those detected (Fig. S5). Medicines detected at all seven sites included antibiotics, sulfamethoxazole and trimethoprim; the cough medicine, guaifenesin; the diabetes medicine, metformin; the influenza medicine, pseudoephedrine; the CHBB drugs, adenosine, atenolol, ephedrine and practolol; the muscle relaxant, methocarbamol; the NSAID, diclofenac and the painkiller, salicylamide. The common detection of sulfamethoxazole agrees with the findings of Faleye et al. (2018), where this was the most commonly found antibiotic in surface waters in Africa. As expected, for surface and wastewaters in South Africa (Abafe et al. 2018; Mosekiemang et al. 2019), we frequently found two antiretroviral drugs (lopinavir and ritonavir). Other antiretroviral drugs (e.g. efavirenz) may have been present, but due to their physico-chemical properties, they are unlikely to be sequestered by the HLB-L material or be sorbed into the PES membrane.

The Hennops ds site had the highest number of medicines detected (64), followed by N14 (61 detections). The least contaminated site was the Diepsloot (34 medicines detected). Diepsloot is a poor community; hence, they may not be able to afford to buy significant amounts of medicine. As 57% of the medicines detected are scheduled above S2 (Table S3), their purchase would require the extra expense of consulting a qualified medical professional in order to obtain a prescription.

To the authors’ knowledge, 83 medicines have been reported for the first time as pollutants in surface waters in South Africa. These include new classes such as anaesthetics, laxatives, hypoactive sexual desire disorder drugs and malaria drugs. Of the seven antihistamine medicines detected, only fexofenadine has been reported previously by Archer et al. (2017b). Likewise, of the 30 antihypotensive and beta-blocking drugs found, only atenolol, ephedrine, irbesartan and valsartan have been reported (Archer et al. 2017a).

### Psychotropic drugs

A total of 52 psychotropic drugs were detected in the Hennops and Jukskei Rivers (Table 2). Psychoactive stimulants were the most frequently detected (33%) (Fig. S6). Several illicit drugs of abuse, e.g. methylenedioxyamphetamine (MDA), 3,4-methylenedioxymethamphetamine (MDMA) and methamphetamine were found. Only 58% of the psychoactive stimulants detected were registered with SAHPRA (30 October 2018). This highlights that there is a high incidence of drug abuse among populations in the upstream areas of the sampling sites. The N14 and Buccleuch sites recorded the highest number (8) of psychoactive stimulants (Table 2). The opioid analgesic, tramadol; the epilepsy drugs, carbamazepine and lamotrigine; the tranquiliser, meprobamate and the CNS depressant drug, methaqualone were detected at all seven sampling sites. Other compounds frequently detected were the antidepressant, venlafaxine and the anticonvulsant epilepsy drug, oxcarbazepine.

Of the psychotropic drugs detected in this study, only two antidepressants (fluoxetine and venlafaxine), one opioid analgesic (tramadol), one psychotic stimulant (amphetamine) and one epilepsy drug have previously been reported by Archer et al. (2017b) and Rimayi et al. (2018a). The cannabis-related compound tetrahydrocannabinol and seven tranquilisers/sedatives are reported for the first time in South African surface waters.

### Metabolites

Human metabolism of xenobiotics is mediated through gene expression by a variety of cytochrome P-450 oxidative enzymes (Buckhout and Thimm 2003) to yield a number of generally more water soluble metabolites. Metabolites of illicit drugs accounted for majority (43%) of the compounds detected. The metabolites of both medicines and nicotine each accounted for 13% of the total. The number (7 to 14 compounds) of metabolites detected was fairly consistent (Table 3). Benzoylecgonine and norcocaine which are metabolites of cocaine, O-desmethylnortramadol and O-desmethyltramadol, both metabolites of tramadol and O-desmethylvenlafaxine, a metabolite of venlafaxine was detected at all seven sampling sites. This indicates a prevalent use of these parent drugs by populations in areas upstream of all the sampling sites and also that their metabolites are stable in the harsh environmental conditions. Of the 23 metabolites detected in the catchment, only cotinine and desvenlafaxine have been reported previously in South African waters (Archer et al. 2017b).

### CNS stimulants

Cocaine was detected in five of the seven sites, indicating its widespread use by the population in the catchment, with the exception of downstream of the Diepsloot and Hennops ds sites (Table 4). Methedrone, 4-MePPP, 4-methylbuphedrone and ethylcathinone are considered illicit recreational drugs and together with cocaine, made up 31% of CNS stimulants detected. Even though the major use of cocaine is generally considered illicit, it is, however, registered by SAHPRA as of 30 October 2018 for some other medical uses. Caffeine and 2-phenethylamine which are found in foods and beverages were detected in all seven sites (Table 4). Only two of the 16 CNS stimulants (caffeine and nicotine) have been found previously in South African surface water by various authors (Archer et al. 2017a, b; Matongo et al. 2015a, b; Wood et al. 2015; Agunbiade and Moodley 2014).

### Pesticides and disinfectants

Pesticides generally include acaricides, fungicides, herbicides, insecticides and vermicides (Settimi et al. 2016). Benzododecinium (a personal care product used as an antiseptic and disinfectant (Richter et al. 2016)), the fungicides, fluconazole and griseofulvin and the insecticide DEET were detected at all sites, indicating their widespread use (Table 5). DEET has been detected previously in surface water around Pretoria (Naude et al. 2015) and prometryn has been found by Rimayi et al. (2018b) in the catchment. Fungicides were the most frequently detected class, making up 37% of the pesticide compounds. Herbicides and insecticides both make up 18% of the total pesticides detected (Fig. S7).

### Food components and additives

Vitamins (biotin (vitamin B7), nicotinamide (vitamin B3) and pyridoxine (vitamin B6)) were the most abundant group detected in the catchment. Nicotinamide and harman, a component of spices, were the most frequently detected food components/additives (Table 6). Artificial sweeteners were the second most abundant compound found. Other food components found consisted of amino acids and components of chocolate and chillies. To the authors’ knowledge, none of these groups has been detected as emerging pollutants in surface water in South Africa.

### Peak intensity–based prioritisation

**Fig. 3:**
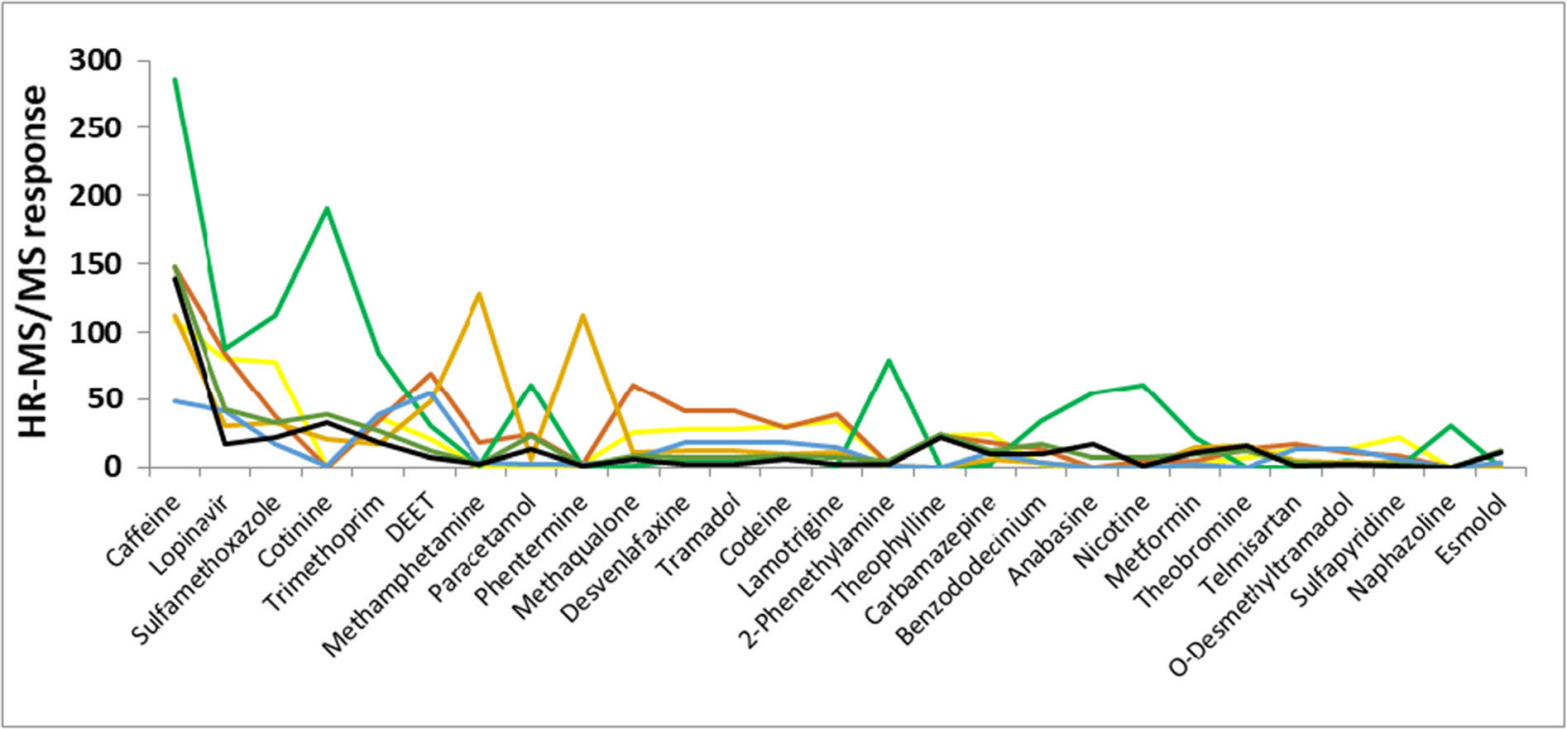
The most significant EP peak intensities (× 10^5^) (based on the 12 highest EP peak intensities per site). Key: Hennops ds (yellow line), N14 (brown line), Diepsloot (green line), Farmall (violet line), Sunninghill (blue line), Midrand (orange line) and Buccleuch (black line)

The five most abundant EPs found, based on relative peak intensity, were caffeine > lopinvar > sulfamethoxazole > cotinine > trimethoprim. These five EPs had the highest peak intensities at all seven sites with caffeine, lopinvar, sulfamethoxazole and trimethoprim being detected at all sites (Fig. 3). The Diepsloot site showed high peak intensities for caffeine, cotinine, 2-phenethylamine, anabasine and nicotine. The most significant peak for the Farmall site was for methamphetamine, and the most significant peak for the Sunninghill site was DEET (Fig. 3). Our data are in general in agreement to the work of Fekadu et al. (2019) who surveyed the frequency of occurrence of pharmaceutical compounds in freshwater aquatic environments in Africa. It should be noted that the peak intensities obtained are influenced by the uptake rate of the compound by the sampler and ionisation efficiency during analysis. These two effects could have influenced the prioritisation procedure.

### Use of Chemcatcher® samplers in qualitative risk assessment studies

This study has shown that the Chemcatcher® is a valuable tool for enabling the screening of emerging pollutants. The use of the passive sampler helps to give lower analytical limits of detection and enhances the potential of identifying episodic contamination events that would otherwise be missed when using spot sampling. Using passive sampling and high-resolution time-of-flight screening, we found 183 new EPs in surface waters in South Africa (Tables 1,2, 3, 4, 5 and 6). From this work and other studies (Amdany et al. 2014b, Archer et al. (2017b), Agunbiade and Moodley (2014, 2016), Madikizela et al. (2017a); Matongo et al. (2015a, b); Rimayi et al. (2018a); Wood et al. (2017)) undertaken at different water bodies in South Africa, we propose a ‘watch list’ of 51 EPs of priority concern (Table S4). This hierarchical listing is based on the frequency of detection and relative peak intensity of the different EPs. The antibiotic, azithromycin, is included in the recent European Union’s Water Framework Directive ‘watch list’ of EPs (Sousa et al. 2019). Ten of the EPs (carbamazepine, fluconazole, ibuprofen, italopram, losartan, naproxen, oxazepam, sulfamethoxazole, tramadol and trimethoprim) in our list have also been recommended by the Swedish Medical Products Agency for future inclusion in the European Union’s Water Framework Directive ‘watch list’ for EPs (Vieno et al. 2017).

## Conclusions

To our knowledge, this is the first time that passive sampling in combination with a liquid chromatography high-resolution mass spectrometry–screening approach has been used to investigate the occurrence of EPs in water bodies in South Africa. The analytical workflow was able to identify 219 compounds with confidence in the Hennops and Jukskei Rivers. A total of 83 medicines, 47 psychotropic drugs and 22 drug metabolites were identified for the first time in surface waters in South Africa. Medicines and psychotropic drugs made up the majority of EPs detected in the catchment. From this and the related work and of others, we have been able to propose a ‘watch list’ of chemicals (33 medicines, 11 psychotropic drugs, 3 CNS stimulants and 4 pesticides) that should be further investigated in future water quality–monitoring programmes within South Africa. Future work should encompass the target analysis of the substances, so that their environmental concentrations can be ascertained and proper risk assessments be formulated and guide future environmental and human health research directions (Gwenzi and Chaukura 2018). Such data could also be used to develop hydro-chemical models for this wide class of pollutants (Wanda et al. 2017. If passive sampling is also to be used in this exercise, it will also necessitate the prior measurement of the sampler uptake rates for these key compounds.

It was evident that the whole catchment is under pressure from significant pollution as reflected in the generally high TDS and low DO values recorded at the seven field sites. The pollution arises from both treated/partially treated sewage effluent from WWTPs as well as direct human and other unregulated inputs. The Jukskei River has many point and diffuse pollution points, which makes its environmental remediation challenging. The Hennops River has the Sunderland Ridge WWTS as the single major pollution source. It, therefore, has a good potential of remediation and for achieving a ‘good’ ecological status if this treatment works and can be upgraded to cope with the high sewage influent volumes that it now has to handle.

## Electronic supplementary material

ESM 1 (DOC 8.40 mb)
