# Supplementary material for: Use of the Chemcatcher® passive sampler and time-of-flight mass spectrometry to screen for emerging pollutants in rivers in Gauteng Province of South Africa
Source: Environ Monit Assess. 2019 May 21;191(6):388. doi: 10.1007/s10661-019-7515-z (PMC6529598; doi:10.1007/s10661-019-7515-z)
Supplement: Supplementary file 1 — (DOC 8.40 mb) [file 10661_2019_7515_MOESM1_ESM.doc]

**Electronic Supplementary Material**

**Use of the Chemcatcher® passive sampler and time-of-flight-mass spectrometry to screen for emerging pollutants in rivers in the Gauteng Province of South Africa**

**Cornelius Rimayi^1^*, Luke Chimuka^2^, Anthony Gravell^3^, Gary R. Fones^4^ and Graham A. Mills^5^**

^1^Department of Water and Sanitation, Resource Quality Information Services (RQIS), Roodeplaat, P. Bag X313, 0001 Pretoria, South Africa

^2^University of the Witwatersrand, School of Chemistry, P. Bag 3, Wits, 2050 Johannesburg, South Africa

^3^Natural Resources Wales, NRW Analytical Services at Swansea University, Swansea University, Faraday Building, Singleton Campus, Swansea, SA2 8PP, UK

^4^School of Earth and Environmental Sciences, University of Portsmouth, Burnaby Road, Portsmouth, PO1 3QL, UK

^5^School of Pharmacy and Biomedical Sciences, University of Portsmouth, White Swan Road, Portsmouth, PO1 2DT, UK

*To whom all correspondence should be addressed.

Phone number: ++271 2808 9500

e-mail: rimayic@dws.gov.za

**Further details of the Jukskei River field sites**

The southernmost downstream Jukskei River field site, **Buccleuch**, is located on the first tributary and lies downstream of the Alexandra Park Township whose municipal sewage facilities are overloaded with up to five times the waste volume it was originally designed for. This situation, therefore, results in frequent unabated raw sewage overflows into the Jukskei River. In addition, upstream of the **Buccleuch** site there numerous informal riverside settlements located on the banks of the river with no municipal sewage facilities. The riverbank communities often use the Jukskei River for their garbage and human waste disposal.

The **Midrand** field site is located 4 km directly downstream of the Buccleuch site and is located adjacent to suburban residential area where there are much lower levels of sewage pollution incidences, but nevertheless, this site affected by pollution from downstream.

The **Sunninghill** field site located on the second tributary, lies in the affluent prime municipal area of Sandton, a well-developed modern suburb surrounded by shopping malls and townhouses. It was selected for comparison of the water quality to sites lying downstream of the poor communities i.e. the Buccleuch and Diepsloot field sites.

The **Farmall** field site is located on a third tributary on the outskirts of the City of Johannesburg. The site lies downstream of prime suburban areas, particularly Randpark located 18 km upstream and Strydompark located 15 km upstream.

The **Diesploot** field site lies in the fourth Jukskei River tributary and consists of what appears as a river of sewage flowing through the Diepsloot informal settlement.

The **N14** site lies downstream of the Buccleuch, Midrand, Sunninghill, Farmall and Diesploot sites (all the other Jukskei River sampling sites). It also lies downstream of the northern wastewater treatment works (WWTW), a large municipal sewage treatment plant.

The GPS coordinates of the field sampling points are shown in Table S1.

**Table S1** GPS coordinates of field sites on the Hennops River and Jukskei River.

| Field Site | Latitude (South) | Longitude (East) |
| --- | --- | --- |
| **Hennops River**  Hennops ds | -25.82701° | 28.10453° |
| **Jukskei River**  N14 | -25.94933° | 27.95878° |
| Diepsloot | -25.94709° | 28.00066° |
| Farmall | -25.97820° | 27.96212° |
| Sunninghill | -26.02791° | 28.05872° |
| Midrand | -26.03139° | 28.11222° |
| Buccleuch | -26.05754° | 28.10410° |

**Table S2** Total dissolved solids (TDS) and dissolved oxygen measurements taken at the seven field sites at the deployment and retrieval of the Chemcatcher® passive samplers.

| **Sampling site** | **TDS at**  **deployment**  **(g L^-1^)** | **TDS at retrieval**  **(g L^-1^)** | **DO at deployment (mg L^-1^)** | **DO at retrieval (mg L^-1^)** |
| --- | --- | --- | --- | --- |
| Hennops ds | 1.02 | 0.67 | 5.18 | 0.57 |
| N14 | 0.46 | 0.43 | 7.19 | 6.37 |
| Diepsloot | 0.62 | 0.58 | 3.09 | 2.00 |
| Farmall | 0.31 | 0.37 | 8.66 | 7.89 |
| Sunninghill | 0.26 | 0.27 | 8.99 | 9.53 |
| Midrand | 0.46 | 0.40 | 6.27 | 5.91 |
| Buccleuch | 0.35 | 0.37 | 3.60 | 3.23 |

**Table S3** Scheduling of medicines in South Africa according to the South African Health Products Regulatory Authority (SAHPRA).

| Schedule | Availability |
| --- | --- |
| 0 | Unregulated, available on the shelves in general shops for self medication |
| 1 | Over the counter in a pharmacy |
| 2 | Over the counter in a pharmacy. Sale record must be kept |
| 3 | Prescription only, allowed to repeat for 6 months. Available from the dispensary in the pharmacy |
| 4 | Prescription only, allowed to repeat for 6 months. Available from the dispensary in the pharmacy |
| 5 | Prescription only, repeats stipulated. Available from the dispensary in the pharmacy |
| 6 | Prescription only, therapeutic narcotics. |
| 7 | Controlled substance |
| 8 | Strictly controlled substances |

**Table S4** Proposed hierarchical ‘watch list’ of emerging pollutants for surface water in South Africa separated by different classes of substance.

| **Medicines** | **Psychotropic drugs** | **CNS stimulants** | **Pesticides** | |  | |
| --- | --- | --- | --- | --- | --- | --- |
| Acetaminophen | Citalopram | Caffeine | Benzododecinium | |  | |
| Adenosine | Hydrocodone | Nicotine | DEET | |  | |
| Atenolol | Lamotrigine | 2-Phenethylamine | Fluconazole | |  | |
| Azithromycin | Levetiracetam |  | Griseofulvin | |  | |
| Cetirizine | Meprobamate |  |  | |  | |
| Codeine | Methaqualone |  |  | |  | |
| Diclofenac | Oxazepam |  |  | |  | |
| Ephedrine | Oxcarbazepine |  |  | |  | |
| Efavirenz | Tramadol |  | |  | |  |
| Fexofenadine | Venlafaxine |  | |  | |  |
| Guaifenesin |  |  | |  | |  |
| Lidocaine |  |  | |  | |  |
| Ibuprofen |  |  | |  | |  |
| Irbesartan |  |  | |  | |  |
| Lopinavir |  |  | |  | |  |
| Losartan |  |  | |  | |  |
| Metformin |  |  | |  | |  |
| Methocarbamol |  |  | |  | |  |
| Naproxen |  |  | |  | |  |
| Nevirapine |  |  | |  | |  |
| Norephedrine |  |  | |  | |  |
| Practolol |  |  | |  | |  |
| Proguanil |  |  | |  | |  |
| Pseudoephedrine |  |  | |  | |  |
| Ritonavir |  |  | |  | |  |
| Salicylamide |  |  | |  | |  |
| Sotalol |  |  | |  | |  |
| Sulfamethoxazole |  |  | |  | |  |
| Sulfapyridine |  |  | |  | |  |
| Telmisartan |  |  | |  | |  |
| Theophylline |  |  | |  | |  |
| Trimethoprim |  |  | |  | |  |
| Valsartan |  |  | |  | |  |


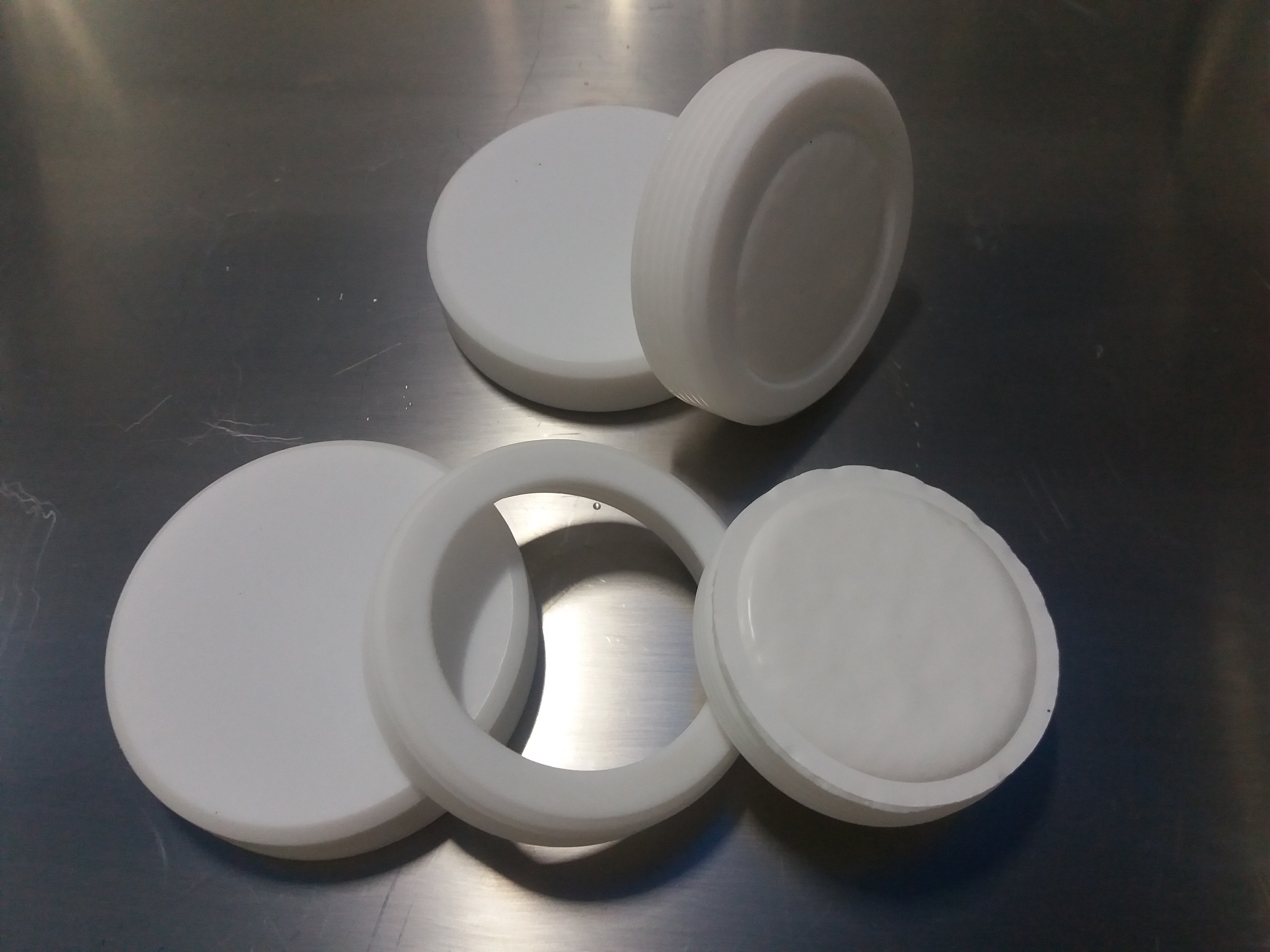


**Fig. S1** Three component Chemcatcher® bodies (top assembled and bottom dissembled).


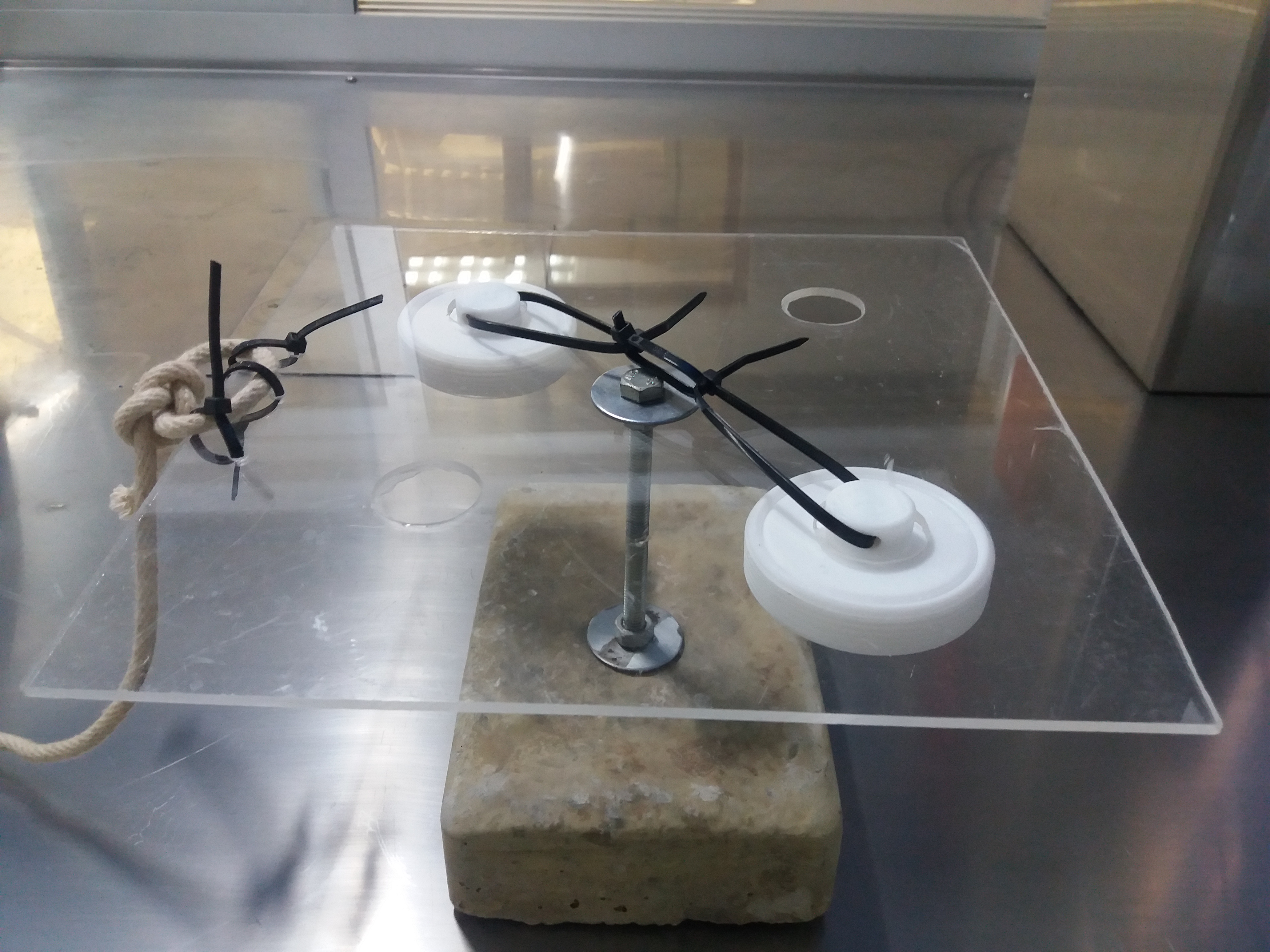


**Fig. S2** Chemcatcher® deployment rig (here, two Chemcatcher® samplers were mounted on a Perspex sheet, supported on a brick as a weight)


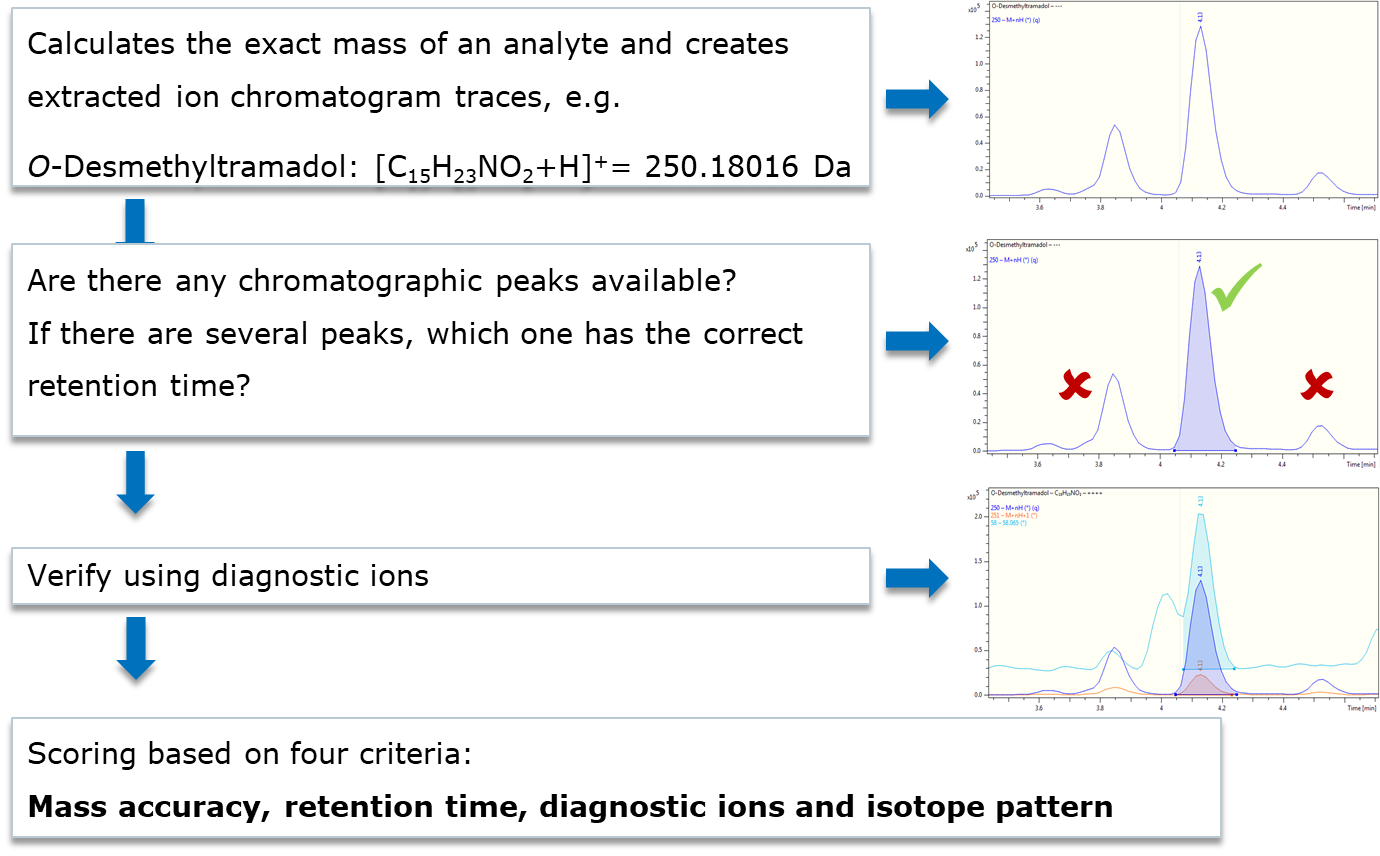


**Fig. S3** HR-LC-MS/MS screening workflow – processing of data.


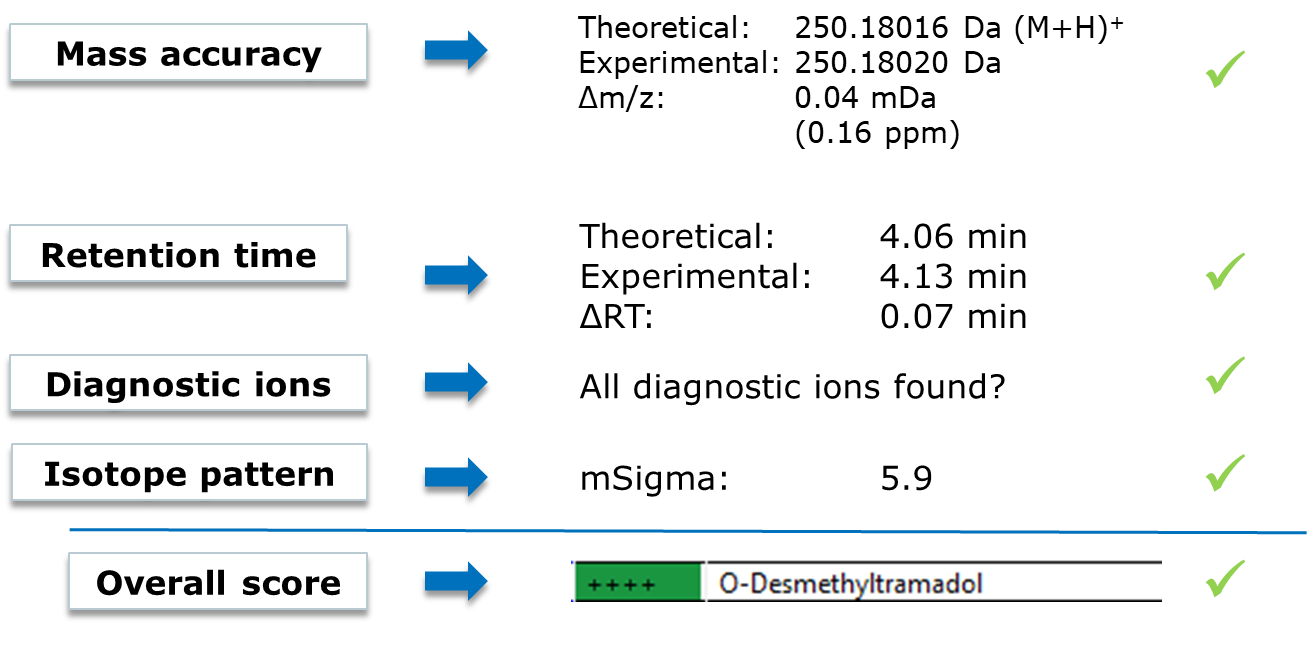


**Fig. S4** HR-LC-MS/MS screening workflow – scoring of data.


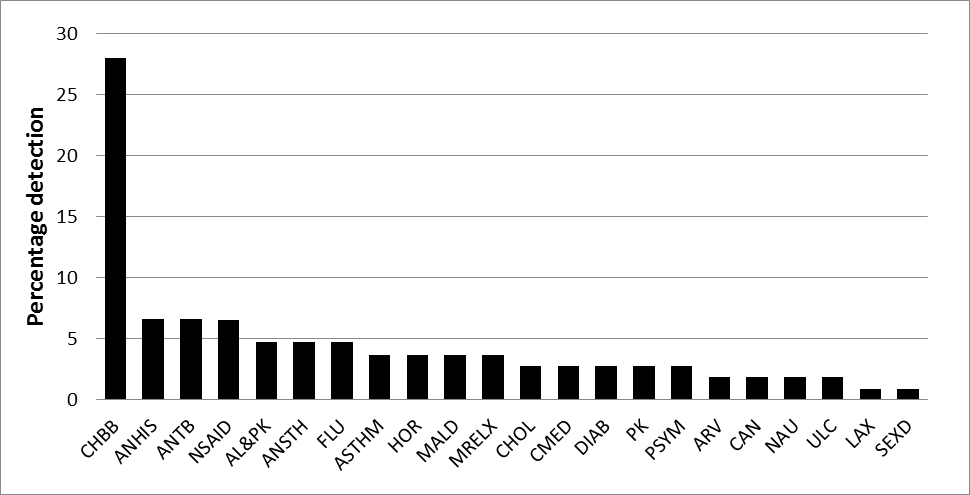


Key

| CHBB | Cardiac and antihypertensive agent/Beta blocking drug | CHOL | Hypercholesterolemia (cholesterol) drug |
| --- | --- | --- | --- |
| ANHIS | Antihistamine medication | CMED | Cough medicine |
| ANTB | Antibiotic drug | DIAB | Diabetes drug |
| NSAID | Nonsteroidal anti-inflammatory drug | PK | Pain killer |
| AL&PK | Alzheimer's disease, Arthritis and Parkinson's disease drug | PSYM | Antipsychotic medication |
| ANSTH | Anaesthetic drug | ARV | Antiretroviral drug |
| FLU | Influenza medication | CAN | Cancer drug |
| ASTHM | Asthma drug | NAU | Nausea drug |
| HOR | Hormone | ULC | Peptic ulcer medication |
| MALD | Malaria drug | LAX | Laxative |
| MRELX | Muscle relaxant | SEXD | Hypoactive sexual desire disorder (HSDD) drug |

**Fig. S5** Distribution of medicines found in the Hennops and Jukskei Rivers.


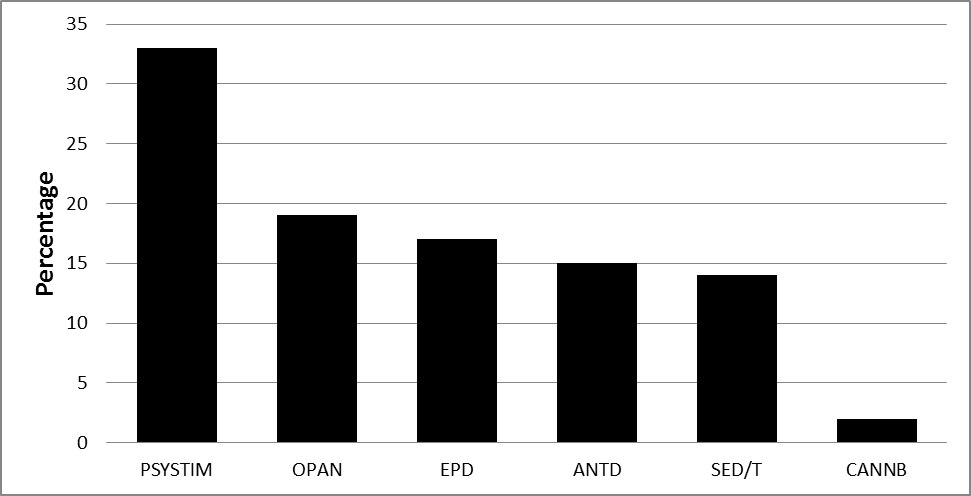


Key

| PSYSTIM | Psychocactive stimulant |
| --- | --- |
| OPAN | Opioid analgesic |
| EPD | Epilepsy and anticonvulsant drug |
| ANTD | Antidepressant |
| SED/T | Sedative/Tranquiliser drug |
| CANNB | Cannabis |

**Fig. S6** Psychotropic drugs detected in the Hennops and Jukskei Rivers.


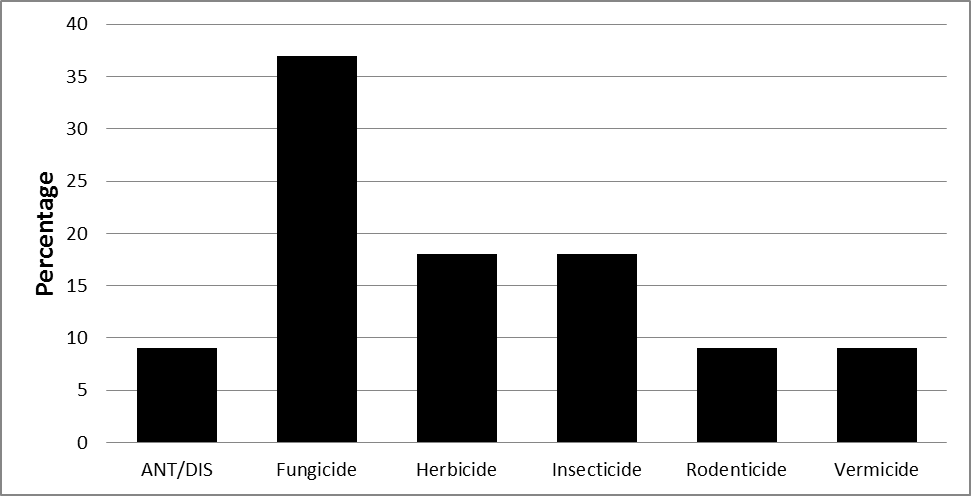


Key

ANT/DIS Antiseptic/disinfectant

**Fig. S7** Pesticides and disinfectants detected in the Hennops and Jukskei Rivers.
